# Glutamic-oxaloacetic transaminase 1 regulates adipocyte differentiation by altering nicotinamide adenine dinucleotide phosphate content

**DOI:** 10.5713/ab.21.0174

**Published:** 2021-08-25

**Authors:** Yang Yang, Zhimin Cheng, Wanfeng Zhang, Wei Hei, Chang Lu, Chunbo Cai, Yan Zhao, Pengfei Gao, Xiaohong Guo, Guoqing Cao, Bugao Li

**Affiliations:** 1 College of Animal Science, Shanxi Agricultural University, Taigu 030801, China; 2 Shanxi Academy of Advanced Research and Innovation, Taiyuan 030032, China

**Keywords:** Adipogenesis, Glutamic-oxaloacetic Transaminase 1 (GOT1), Intramuscular Fat, Lipid Metabolism, Nicotinamide Adenine Dinucleotide Phosphate (NADPH)

## Abstract

**Objective:**

This study was performed to examine whether the porcine glutamic-oxaloacetic transaminase 1 (*GOT1*) gene has important functions in regulating adipocyte differentiation.

**Methods:**

Porcine *GOT1* knockout and overexpression vectors were constructed and transfected into the mouse adipogenic 3T3-L1 cells. Lipid droplets levels were measured after 8 days of differentiation. The mechanisms through which GOT1 participated in lipid deposition were examined by measuring the expression of malate dehydrogenase 1 (*MDH1*) and malic enzyme (*ME1*) and the cellular nicotinamide adenine dinucleotide phosphate (NADPH) content.

**Results:**

*GOT1* knockout significantly decreased lipid deposition in the 3T3-L1 cells (p< 0.01), whereas *GOT1* overexpression significantly increased lipid accumulation (p<0.01). At the same time, *GOT1* knockout significantly decreased the NADPH content and the expression of *MDH1* and *ME1* in the 3T3-L1 cells. Overexpression of *GOT1* significantly increased the NADPH content and the expression of *MDH1* and *ME1*, suggesting that GOT1 regulated adipocyte differentiation by altering the NADPH content.

**Conclusion:**

The results preliminarily revealed the effector mechanisms of *GOT1* in regulating adipose differentiation. Thus, a theoretical basis is provided for improving the quality of pork and studies on diseases associated with lipid metabolism.

## INTRODUCTION

Pigs are highly similar to humans in genetic and metabolic mechanisms. Therefore, the use of a pig as a model to study metabolism and obesity is significant [1]. The adipose tissue is the largest energy storage tissue in the body, and adipocytes can be derived from the differentiation of pluripotent mesenchymal stem cells [2]. Adipocyte differentiation is a process by which lipid droplets continuously accumulate in the cells, ultimately forming mature adipocytes [3]. The deposition of intramuscular fat (IMF) causes muscles to form marble patterns in meat, and IMF content is one of the important factors that affect the muscle quality. IMF is closely associated with muscle tenderness, meat colour and meat flavour [4]. Amino acids are not only the basic building blocks of proteins, but they can also be converted to lipids and carbohydrates. Numerous studies have also shown that amino acids play an important regulatory role in lipid metabolism [5].

The Mashen pig is an indigenous breed in the Shanxi province and characterised as an obese breed. By contrast, the Large White pig is considered as a lean breed. These two breeds show significant differences in lipid deposition, the IMF content of the Mashen pig was significantly higher than that of the Large White pig (p<0.01) [6]. In our previous studies, we showed that the levels of flavour amino acids, such as aspartate and glutamate, at the longissimus muscle of 6-month-old Mashen pigs were higher than those of Large White pigs. Glutamic-oxaloacetic transaminase (GOT) connects the two abovementioned amino acids. GOT was mainly found as cytoplasmic GOT and mitochondrial GOT, which were encoded by the *GOT1* and *GOT2* genes, respectively. RNA-seq results of the longissimus muscles from the 6-month-old Mashen and Large White pigs showed substantial differences in the expression levels of *GOT1*, malate dehydrogenase 1 (*MDH1*) and pyruvate dehydrogenase alpha 1 (*PDHA1*). Furthermore, these genes were enriched in the amino acid metabolic pathways. Through bioinformatics analysis, we found a key candidate gene, *GOT1*, which was significantly enriched in the leucine, aspartate and glutamate pathways [7].

GOT1 is a pyridoxal phosphate-dependent enzyme that participates in a reversible reaction in the cytoplasm where aspartate and α-ketoglutarate are used to synthesise glutamate and oxaloacetate [8]. This enzyme also has an important role in maintaining the cellular redox balance, promoting tumour cell proliferation and regulating amino acid metabolism [9]. Studies on colon cancer cell lines have found that overexpression of *GOT1* caused cells to synthesize nicotinamide adenine dinucleotide phosphate (NADPH) to regulate the levels of reactive oxygen species and promote tumour growth. In addition, treating cells with aminooxyacetate to inhibit GOT1 could induce apoptosis in tumour cells, and targeting GOT1 could selectively kill tumour cells [10,11]. Knocking out the *GOT1* gene can inhibit the glutamine metabolic pathway and decrease the NADPH synthesis, thereby inhibiting cell proliferation [12]. Studies on *GOT1* have mainly focused on tumours, and only a few studies have investigated the participation of *GOT1* in lipid synthesis.

In this study, we attempted to determine the role of GOT1 in adipocyte differentiation. We constructed porcine *GOT1* gene knockout and overexpression vectors and transfected these vectors into 3T3-L1 cells. We used quantitative reverse-transcription polymerase chain reaction (qRT-PCR), Western blot and oil red-O staining (ORO) to investigate the effects of the *GOT1* gene on the adipogenesis in the 3T3-L1 cells and examine the effector mechanisms of the *GOT1* gene in adipocyte differentiation.

## MATERIALS AND METHODS

### Experimental animals and sample collection

The six 6-month-old Mashen pigs and six Large White pigs used in this experiment were obtained from the Datong pig farm and housed under the same conditions. All animal procedures were performed in strict accordance with the Code of Ethics of the World Medical Association (http://ec.europa.eu/environment/chemicals/lab_animals/legislation_en.htm). Experimental protocols were approved by the Animal Ethics Committee of Shanxi Agricultural University (Shanxi, China). The number of Ethics Committee agreement is SXAU-EAW-P002003. Pigs were euthanised according to standard slaughtering procedures. After slaughter, the heart, liver, subcutaneous fat at the back, spleen, kidneys, small intestine, cerebellum, longissimus muscle, psoas major muscles and biceps femoris muscles were collected and snapped frozen in liquid nitrogen before they were transferred to a freezer at −80°C for storage.

### Materials

The restriction endonuclease *Bsm*B I and T4 DNA ligase were purchased from NEB, Inc. (Ipswich, MA, USA). The gel recovery kit, PrimeScript RT reagent Kit with gDNA Eraser reverse transcription kit, SYBR Premix ExTaq RT-PCR kit and competent cells were purchased from TaKaRa Bio Japan, Inc. (Tokyo, Japan). The plasmid isolation kit was purchased from Omega Bio-tek, Inc. (Norcross, GA, USA). Rabbit GOT1 primary antibodies (bs-3977R), rabbit MDH1 primary antibodies (bs-3996R) and rabbit β-actin primary antibodies (bs-0061R) were purchased from Bioss, Inc. (Woburn, MA, USA). Goat anti-rabbit fluorescent secondary antibodies were purchased from LI-COR Biosciences, Inc. (Lincoln, NE, USA). Penicillin–streptomycin and NADP/NADPH assay kits were purchased from Solarbio Science & Technology Co., Ltd. (Beijing, China). Low-glucose Dulbecco’s modified eagle medium (DMEM) and foetal bovine serum (FBS) were purchased from Gibco, Inc. (San Diego, CA, USA).

### sgRNA design and synthesis

The cDNA for porcine *GOT1* (Genbank accession no: NM_213927.1) was inputted into the website http://crispr.dbcls.jp/ for the sgRNA design. Three sgRNA sequences with high score and specificity were designated as sg1, sg2, and sg3 (Table 1). A *BsmB* I site was added at the 5′ end. All sgRNA sequences were synthesised by Sangon Biotech Co., Ltd. (Shanghai, China).

### Construction and identification of the Cas9-GOT1 knockout vector

Diluted sgRNA (10 μmoL/L) was annealed based on the annealing program (93°C for 5 min, followed by a 1°C decrease every min to 25°C for a total of 68 cycles). The annealing solution was 2 μL of 10×buffer, 2 μL each of the upstream and downstream primers for sgRNA and ddH_2_O, which was added for a final volume of 20 μL. To cleave the Cas9 vector backbone, *Bsm*B I cleavage was carried out according to the following conditions: at 55°C for 2 h and then at 80°C for 10 min. The enzyme cleavage solution was 1 μL of 10×buffer, 500 ng of plasmid DNA, 1 μL of *Bsm*B I (10,000 U/mL) and ddH_2_O, which was added for a final volume of 10 μL. T4 ligase was used to ligate the cleaved Cas9 vector and the annealed sgRNA sequence to construct the Cas9-GOT1 knockout vector. The ligated product was transformed into DH5α competent cells, and the plasmids were extracted for sequencing and identification. Recombinant plasmids that were confirmed to be successful by sequencing were designated as GOT1_KO1, GOT1_KO2, and GOT1_KO3. Endotoxin-free plasmid extraction kits were used to extract the three plasmids for use in subsequent experiments.

### Detection of the knockout efficiency of the Cas9-GOT1 vector

PK15 cells were subcultured onto a 10 cm culture dish and cultured with low-glucose DMEM containing 10% FBS and 1% penicillin–streptomycin at 37°C and 5% CO_2_. An electroporator from Lonza (Cologne, Germany) was employed for transfection. When cells grew to 70% to 80% confluence, 0.25% trypsin solution was used for digestion at 37°C. A total of 4×10^5^ cells were diluted to 100 μL of electroporation solution, and 2 μg of the above plasmid was added. Then, electroporation was carried out based on the program (DS-130) provided by Lonza (Germany). After 12 h of transfection, the cells were observed under a fluorescence microscope (Leica, Germany), and the transfection efficiency was determined. Cells that were transfected with empty plasmids were set as the negative control group, whereas those transfected with GOT1_KO1, GOT1_KO2, and GOT1_KO3 recombinant plasmids were set as the knockout groups. After 1 week of screening with puromycin (1 μg/mL), the culture medium was changed, and proteins were extracted after the cells were confluent. Western blot analysis was then used to measure the effects of *GOT1* gene knockout.

### Construction and identification of the over-GOT1 expression vector

The total RNA was extracted from porcine muscle tissues and reverse transcribed into cDNA. Upstream and downstream primers were designed based on the *GOT1* cDNA (Genbank Accession No. NM_213927.1): forward primer: F1: 5′-CCCAAGCTTGCCACCATGGCACCTCCATCA GTCTTTGCCG-3′ (*Hin*d III site was underlined, and the red loci were enhancers), and reverse primer: R1: 5′-CCG GAATTCTCACTGGATTTTGGTGACAGCTTCA-3′ (*Eco*R I site was underlined) were used to amplify the full-length coding sequences (CDS). Then, a gel recovery kit was used for purification.

*Hin*d III and *Eco*R I enzymes were used to cleave the purified products and the empty pCDNA-3.1-EGFP vector. Then, T4 ligase was used to ligate the cleaved purified product and the pCDNA-3.1-EGFP vector to finally obtain the *GOT1* overexpression vector, pCDNA-3.1-EGFP-GOT1. The ligation product was transformed into DH5α competent cells. Endotoxin-free plasmid extraction kits were used to extract the plasmids, which were used for subsequent experiments after the sequences were verified.

### Transfection and induced differentiation of the 3T3-L1 cells

When the 3T3-L1 cells had grown to 70% to 80% confluence, 0.25% trypsin solution was used for digestion at 37°C. A total of 4×10^5^ cells were diluted in 100 μL of the electroporation solution, and 2 μg of the above plasmid were added. Then, electroporation was performed based on the program (CM-137) provided by Lonza (Germany). After 12 h of transfection, the cells were observed under a fluorescence microscope, and the transfection efficiency was determined. Cells transfected with the empty pCDNA-3.1-EGFP vector were set as the overexpression negative control group (control), whereas the cells transfected with the pCDNA-3.1-EGFP-GOT1 overexpression vector were set as the overexpression group (Over-GOT1). Cells transfected with the empty Cas9 vector were set as the knockout negative control group, whereas cells transfected with GOT1_KO3 plasmid were set as the knockout group. G418 (250 μg/mL) was used to screen the overexpression vector, whereas puromycin (1 μg/mL) was used to screen the knockout vector. After 1 week of screening, the medium was changed to a normal culture medium (low-glucose DMEM + 10% FBS + 1% penicillin–streptomycin). Differentiation was induced after the cells were confluent.

The methods used to induce differentiation of the 3T3-L1 cells in the control and test groups were the same. Differentiation culture medium (high-glucose DMEM + 10% FBS + 1% penicillin-streptomycin + 5 μg/mL insulin + 1 μM dexamethasone + 0.5 mM IBMX) was used for cell culture and changed once every 2 days. After 4 days, the medium was changed to the differentiation maintenance medium (low-glucose DMEM + 10% FBS + 1% penicillin-streptomycin + 5 μg/mL insulin) and changed once every 2 days. The cells were used for subsequent experiments after 8 days of differentiation.

### Oil red-O staining

The ORO staining was employed to examine the 3T3-L1 adipocyte differentiation. Cells that underwent differentiation for 8 days were washed twice with phosphate-buffered saline (PBS) before they were fixed with formaldehyde (10%) for 10 min. The cells were then washed again with PBS and stained with freshly diluted ORO for 15 min. After staining, the cells were washed with PBS and photographed using a microscope (Leica, Germany).

### Quantitative reverse-transcription polymerase chain reaction

Total RNA was extracted from the cells and then reverse transcribed into cDNA. The relative expression of *GOT1*, *MDH1*, and adipogenesis-related genes, peroxisome proliferator activated receptor gamma (*PPARγ*) and fatty acid binding protein 4 (*FABP4*, also known as *ap2*), were measured in the different groups. 18S was used as an internal reference gene. The 10 μL reaction solution consisted of 5 μL of SYBR, 0.2 μL of Rox, 0.15 μL of each upstream and downstream primers, 2 μL of cDNA, and 2.5 μL of ddH_2_O. The reaction conditions were as follows: predenaturation at 95°C for 20 s, followed by 36 cycles of denaturation at 95°C for 20 s, and annealing and extension at 60°C for 20 s. The results were calculated using the 2^−ΔΔCT^ method. Table 2 shows the primer sequences.

### Western blot

The total protein was extracted from the cells in the knockout, overexpression and corresponding control groups. Then, 20 μg of the protein from each group were loaded onto an sodium dodecyl sulfate-polyacrylamide gel before gel electrophoresis was performed. The proteins were transferred to a membrane, blocked and incubated with primary and secondary antibodies. The LICOR imager was used to develop the membranes, and the imager software Image Studio was used to calculate the optical density of the bands.

### Measurement of NADPH levels in cells

The NADP/NADPH ratio assay kit was used to measure NADPH levels in cells. After cells were washed thrice with PBS, the NADP/NADPH extraction buffer was added, and the cells were incubated on ice for 20 min, followed by incubation at room temperature for 10 min. The supernatants were collected, and the absorbance was measured at 590 nm. The NADP/NADPH ratio was used to reflect the relative level of NADPH.

### Statistical analysis

One-way analysis of variance was used to analyse the variance and test the significance for the collected data using the GraphPad Prism 5 software. A difference of p<0.05 was considered to be statistically significant.

## RESULTS

### Construction and screening of *GOT1* knockout vectors

**Figure 1:**
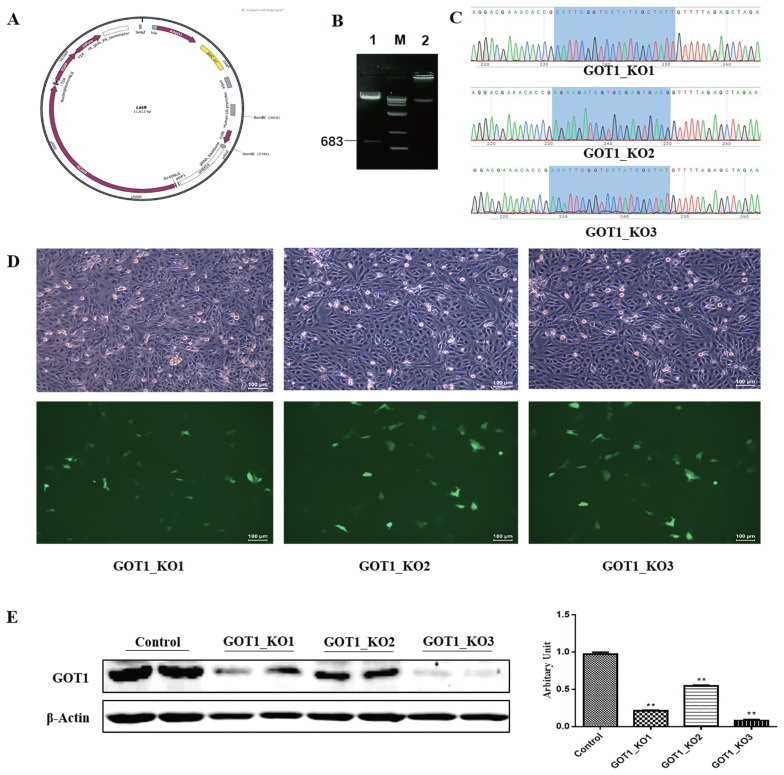
Identification and screening of *GOT1* knockout vectors. (A) A schematic representation of the Cas9 plasmid. (B) Gel electrophoresis image of cleavage of Cas9 plasmid by using *Bsm*B I: M, DL10,000 marker; 1, cleavage of Cas9 plasmid by using *Bsm*B I; 2, Cas9 plasmid. (C) Plasmid sequencing chromatogram, blue-shaded region represented the sgRNA sequence. (D) Fluorescence images of PK15 cells transfected with GOT1_KO1, GOT1_KO2, and GOT1_KO3 plasmids: upper, bright field; lower, fluorescence. (E) Protein levels of GOT1 in the PK15 cells measured by Western blot and quantitative analysis by Image Studio; control is the negative control group, and β-actin was used as a loading control. Data are shown as means±standard error of the mean. *GOT1*, glutamic-oxaloacetic transaminase 1. ** Indicates significant difference from the control (p<0.01).

The synthesised sgRNA sequences underwent annealing before ligation with the *Bsm*B I-linearised Cas9 plasmid (Figure 1A, B). The ligation product was transformed into competent cells. After plating, culture and isolation of single colonies, the plasmids were extracted for sequencing and identification. The results showed that the three recombinant plasmids were successfully constructed (Figure 1C). Green fluorescence could be observed for the negative control and knockout groups under a fluorescence microscope. The transfection efficiency of the PK15 cells was 20% to 30%, indicating that the plasmid transfection was successful (Figure 1D). Puromycin (1 μg/mL) was used to treat the cells for 1 week. After the cells became confluent, the cells were cultured for another 48 h before collection. The total protein was collected from the cells from the different groups for Western blot analysis. The results showed that, compared with the control group, the protein expression levels in the GOT1_KO1, GOT1_KO2, and GOT1_KO3 groups were significantly decreased (p<0.01), and the reduction of GOT1 in the GOT1_KO3 group was the most significant (Figure 1E). This result indicated that the knockout effects of GOT1_KO3 were the most efficient.

### Construction and identification of the *GOT1* overexpression vector

**Figure 2:**
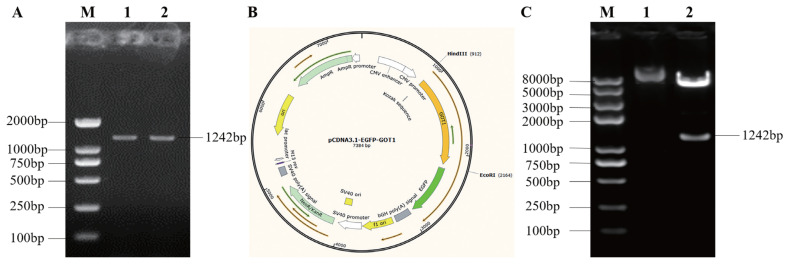
Porcine *GOT1* CDS amplification and construction of the Over-GOT1 expression vector. (A) Gel electrophoresis image of the RT-PCR of the porcine *GOT1* gene: M, DL200 marker; 1, 2, CDS amplification of the porcine *GOT1* gene. (B) Schematic representation of the pCDNA-3.1-EGFP-GOT1 recombinant plasmid. (C) Gel electrophoresis image of the double digestion of pCDNA-3.1-EGFP-GOT1 with *Hin*d III and *Eco*R I: M, Trans2K marker; 1, pCDNA-3.1-EGFP-GOT1 plasmid; 2, double-digested pCDNA-3.1-EGFP-GOT1 plasmid. *GOT1*, glutamic-oxaloacetic transaminase 1; CDS, coding sequences; EGFP, enhanced green fluorescent protein; RT-PCR, quantitative reverse-transcription polymerase chain reaction.

The amplified porcine *GOT1* gene CDS (Figure 2A) was ligated to an empty pCDNA-3.1-EGFP plasmid to obtain the pCDNA-3.1-EGFP-GOT1 recombinant plasmid (Figure 2B). The ligation product was transformed into competent cells. After plating, culture and isolation of the single colonies, plasmids were extracted for sequencing and identification. The results showed that the *GOT1* overexpression plasmid was successfully constructed. Then, *Hin*d III and *Eco*R I were used to cleave the successfully sequenced plasmid and a band of the same size as the PCR product was obtained. This result also showed that the *GOT1* overexpression plasmid was successfully constructed (Figure 2C) and could be used for transfection experiments.

### Effects of *GOT1* knockout and overexpression on adipocyte differentiation

**Figure 3:**
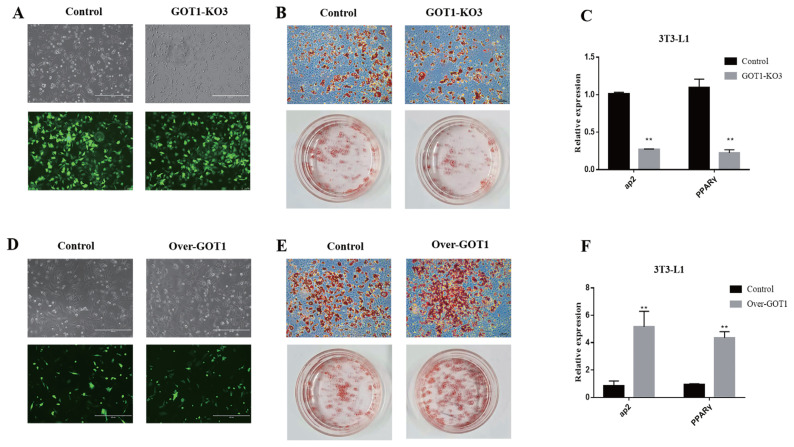
Effects of *GOT1* knockout and overexpression on adipocyte differentiation. (A) Fluorescence images of 3T3-L1 cells transfected with control and GOT1_KO3 plasmids: upper, bright field; lower, fluorescence. (B) Adipogenic phenotypes of 3T3-L1 cells with *GOT1* knockout or control cells after MDI induction for 8 days and were assessed by oil red O (ORO) staining. (C) mRNA levels of *aP2* and *PPARγ* at day 8 as detected by qRT-PCR. (D) Fluorescence images of 3T3-L1 cells transfected with control and *GOT1*_overexpression plasmids: upper, bright field; lower, fluorescence. (E) Adipogenic phenotypes of the 3T3-L1 cells with *GOT1* overexpression or control after MDI induction for 8 days as assessed by ORO staining. (C) mRNA levels of *aP2* and *PPARγ* on day 8 as detected by qRT-PCR. Data are shown as means±standard error of the mean. *GOT1*, glutamic-oxaloacetic transaminase 1; *PPARγ*, peroxisome proliferator activated receptor gamma; qRT-PCR, quantitative reverse-transcription polymerase chain reaction; MDI, 0.5 mM IBMX, 1 μM dexamethasone, and 5 μg/mL insulin. ** Indicates significant difference from the control (p<0.01).

The Cas9 empty plasmid and the knockout plasmid GOT1_KO3 were transfected into the 3T3-L1 cells, and cells were observed after 12 h. Green fluorescence could be observed for the knockout and the negative control groups under a fluorescence microscope. The transfection efficiency was high, showing that plasmid transfection was successful (Figure 3A). Puromycin (1 μg/mL) was used to treat the cells for 1 week. Differentiation was induced after the cells became confluent. After the 3T3-L1 preadipocytes were differentiated (8 days), ORO staining was used to assess the status of lipid accumulation. The results showed a significantly lower lipid accumulation in the cells from the *GOT1* knockout group (Figure 3B). RNA was extracted from the cells of each group for qRT-PCR. The results showed that the mRNA expression levels of *aP2* and *PPARγ* were also significantly decreased (p<0.01) (Figure 3C).

The empty overexpression vector and GOT1 overexpression vector were transfected into the 3T3-L1 cells, and the cells were observed after 12 h. Green fluorescence could be observed for both the overexpression group and the negative control group under a fluorescence microscope. The transfection efficiency was high, showing that the plasmid transfection was successful (Figure 3D). G418 (250 μg/mL) was used to treat the cells for 1 week, and differentiation was induced after the cells were confluent. After differentiation was induced in the cells (8 days), ORO staining was performed. The results showed that lipid accumulation was significantly increased in the cells overexpressing *GOT1* (Figure 3E). The mRNA expression levels of *aP2* and *PPARγ* were also significantly increased (p<0.01) (Figure 3F).

### GOT1 regulation of the adipose cell differentiation by changing the NADPH content

**Figure 4:**
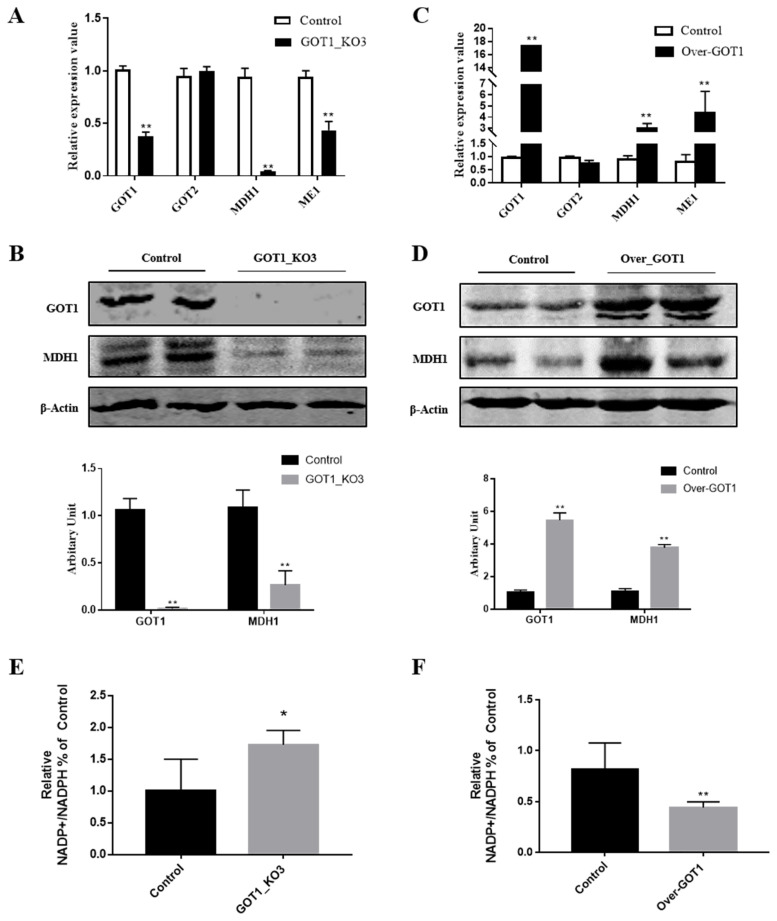
GOT1 regulates adipose cell differentiation by altering the NADPH content. (A, C) mRNA expression levels of *GOT1*, *GOT2*, *MDH1*, and *ME1* in the 3T3-L1 cells after transfection of knockout (A) and overexpression plasmids (C). (B, D) protein levels of GOT1 and MDH1 in the 3T3-L1 cells after transfection of knockout (B) and overexpression plasmids (D). (E, F) NADPH content in the 3T3-L1 cells after transfection of knockout (E) and overexpression plasmids (F). The results are shown as NADP+/NADPH ratios. Data are shown as means±standard error of the mean. *GOT1*, glutamic-oxaloacetic transaminase 1; NADPH, nicotinamide adenine dinucleotide phosphate; *MDH1*, malate dehydrogenase 1; *ME1*, malic enzyme. * Indicate significant difference from the control at p<0.05 and ** p<0.01, respectively.

Aspartate undergoes catalysis by GOT1 in the cytoplasm to become oxaloacetate, which then undergoes MDH1 catalysis to form malate. The NADPH that participates in the catalysis of fatty acid synthesis is synthesised by the malic enzyme (ME1), which catalyses the reaction with malate. After knocking out *GOT1*, we measured the mRNA and protein expression levels of MDH1 and found that they were significantly decreased (p<0.01) (Figure 4A, B). By contrast, overexpressing *GOT1* led to a significant increase in the mRNA and protein expression levels of MDH1 (p<0.01) (Figure 4C, D). Considering that the *GOT1* gene is homologous to *GOT2*, we also measured the expression of the *GOT2* gene but did not observe any changes (Figure 4A, C). We speculate that this phenomenon may be because the regulation of lipid synthesis mainly occurred in the cytoplasm, whereas GOT2 mainly carried out its function in the mitochondrial matrix. Therefore, the expression levels of *GOT2* did not change. After knocking out *GOT1*, the NADPH content in the cells significantly increased (p<0.01) (Figure 4E). Conversely, *GOT1* overexpression caused the NADPH content to notably decrease (p<0.01) (Figure 4F). Combining this result with the MDH1 expression status, this experiment demonstrated that GOT1 regulated adipocyte differentiation by changing the NADPH content.

### Characteristics of *GOT1* expression in the Mashen and Large White pigs

**Figure 5:**
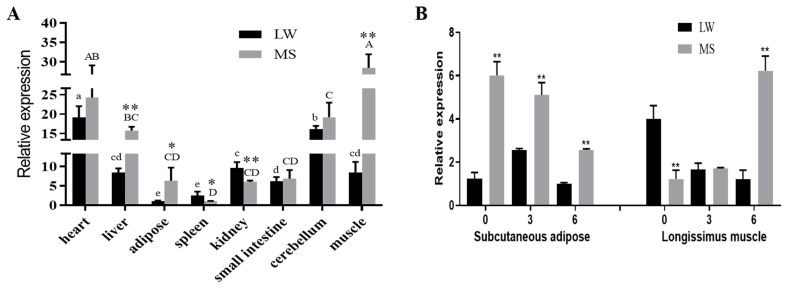
Characteristics of *GOT1* expression in the Mashen and Large White pigs. (A) mRNA expression of *GOT1* in the different tissues in the two breeds of pig; (B) mRNA expression of *GOT1* in the subcutaneous adipose and longissimus muscle in the 0-, 3-, and 6-month-old Large White and Mashen pigs. Data are shown as means±standard error of the mean. * p<0.05 and ** p<0.01 indicate the significant differential expression of *GOT1* within the same tissue between the two breeds of pigs. Different lowercase letters show the significant expression of *GOT1* in the Large White pigs (p<0.05), whereas the uppercase letters show the significant expression of *GOT1* in the Mashen pigs (p<0.05). *GOT1*, glutamic-oxaloacetic transaminase 1.

As shown in Figure 5, the *GOT1* is expressed in the heart, liver, adipose tissue, spleen, kidneys, small intestine, cerebellum and muscle of both breeds. *GOT1* expression was higher in the heart, liver and muscles but lower in the spleen. Higher mRNA levels of *GOT1* were observed in the spleen and kidneys of the Large White pigs than those of the Mashen pigs (p<0.05). However, the mRNA expression levels in the muscles, liver and adipose tissue were all higher in the Mashen pigs than those of the other breed (p<0.05) (Figure 5A).

To study the temporal expression characteristics of *GOT1*, we measured its mRNA expression in the subcutaneous adipose of the 0-, 3-, and 6-month-old Mashen and Large White pigs. The results showed that expression of *GOT1* in the Mashen pigs was remarkably higher than in the Large White pigs at each time point (Figure 5B). Considering that *GOT1* had a significantly higher expression in the muscle of the Mashen pigs and that the IMF in the muscles increased with maturity, we also measured the *GOT1* mRNA levels in the longissimus muscle. Compared with that of the Large White pigs, the mRNA level of *GOT1* in the Mashen pigs was lower at birth (0-month-old) but reversely increased in the 6-month-old Mashen pigs (Figure 5B). These results implied that GOT1 played an important role in the adipose and late stage of IMF formation in Mashen pigs.

## DISCUSSION

Pork is an important food source for humans. Given that the consumer’s preferences and needs continuously change, more and more people tend to choose a better quality of pork, promoting changes in the pork industry [13–15]. The IMF is fat between muscle fibres [16] and is one of the important factors that affect meat quality, because it is closely associated with muscle tenderness, meat colour, and meat flavour [17,18]. In mammals, the intramuscular adipocytes mostly contain triglycerides [19]. Nutrition, environment, genetics and other factors may result in changes in the IMF content. Amino acids can not only act as basic building blocks for proteins but also play an important regulatory role in lipid metabolism [20].

The clustered regularly interspaced short palindromic repeats (CRISPR)-CRISPR-associated protein-9 (Cas9) system is based on the complementary base pairing between a guide RNA and a target DNA to achieve the specific cleavage of genomic DNA. This system is widely used in studies on the modification of genome targets in different animals [21–23]. In this study, we used the website http://crispr.dbcls.jp/ to design sgRNA against the CDS region of the *GOT1* gene. We selected three sequences with the highest scores to construct the knockout vectors. The results showed that the GOT1 protein was highly suppressed in the PK15 cells of the GOT1-KO3 group. This phenomenon demonstrated that, among the three constructed knockout vectors, GOT1-KO3 had the most significant *GOT1* knockout effect. We then transfected this plasmid transfected into the 3T3-L1 cells. Then, the cells were induced to differentiate into mature adipocytes. Compared with the control group, lipid accumulation was significantly inhibited in the *GOT1* knockout group. At the same time, the mRNA expression levels of adipogenesis markers, such as *PPARγ* and *aP2*, were significantly decreased. We also constructed *GOT1* overexpression vectors, which were then transfected into 3T3-L1 cells. We found that GOT1 overexpression significantly promoted lipid accumulation.

To examine the effector mechanisms of GOT1 in lipid accumulation, we measured the expression levels of *MDH1*, which is involved in the malate shuttle. The results showed that they are significantly reduced in the knockout group. However, the expression levels of *MDH1* were significantly increased in the overexpression group. Interestingly, the MDH1 content in the muscle tissues had a significant positive correlation with IMF. MDH1 activity tests in the muscles of both breeds demonstrated that the MDH1 activity in the Mashen pigs was significantly greater than that in the Large White pigs [6]. In a study, the overexpression of MDH1 in the 3T3-L1 cells induced a significant increase in the number of cells undergoing adipogenesis. In addition, the expression levels of the adipogenesis markers, such as *PPARγ* and *C/EBPα*, were also significantly increased when MDH1 was overexpressed [24]. This result indicated that MDH1 could affect meat quality by affecting the IMF content. During the amino acid metabolism, aspartate underwent catalysis by GOT1 in the cytoplasm to become oxaloacetate, which then underwent MDH1 catalysis to form malate. The malate produced in the aforementioned process was catalysed by ME1 to become pyruvate. After pyruvate entered the citric acid cycle, it was oxidised under PDHA1 catalysis to become acetyl-CoA, H^+^, CO_2_, and NADH [25]. Acetyl-CoA from the PDHA1-catalysed reaction is an important raw material for the citric acid cycle and synthesis of the fatty acid and cholesterol [26]. NADPH, which has antioxidant effects, was synthesised by the ME1-catalysed malic acid reaction. NADPH is an important reducing agent that can catalyse the synthesis of fatty acid [27]. The synthetic pathways for intracellular NADPH occurred through the redox branch of the pentose phosphate pathway and the malate shuttle pathway [28]. In the adipocytes, the NADPH produced by the malate shuttle pathway was higher than that produced by the pentose phosphate pathway [29]. A study has shown that MDH1 overexpression could result in more NADPH synthesis [30]. The NADP/NADPH ratio was inversely proportional to the NADPH content, and NADPH was significantly decreased in the knockout group. The NADPH content was significantly increased in the overexpression group. Given that GOT2 is also involved in the malate shuttle, we simultaneously measured the mRNA expression levels of the *GOT2* gene. We found that the mRNA expression levels of *GOT2* did not significantly change in either the knockout or overexpression groups. This phenomenon also indirectly proved that the malate produced in the malate shuttle (in which GOT1 participated) was mostly used in the cytoplasm for NADPH synthesis and not in the mitochondrial matrix. By combining the NADPH synthesis and the above study results, we demonstrated that GOT1 regulated adipocyte differentiation by altering the NADPH content.

Then, we further analysed the spatiotemporal expression characteristics of *GOT1* between the Mashen pigs (naturally obese) and Large White pigs (naturally lean). The results showed that the expression levels of the *GOT1* adipose tissue in the Mashen pigs were significantly higher than in the Large White pigs (p<0.01), which suggested that the high expression of *GOT1* may be a key factor in the higher fat content in the Mashen pigs [6,7]. Interestingly, the mRNA level of *GOT1* in the longissimus muscle of the Mashen pigs was significantly increased at 6 months of age. Combined with the high IMF content in the Mashen pigs, we hypothesized that *GOT1* played an important role in the late stage of fat formation, which resulted in significant differences in the IMF in the later growth of these two pig breeds.

## CONCLUSION

We found a positive role of the amino acid metabolism-associated gene GOT1 in adipogenic differentiation. The function of GOT1 was at least partially mediated by alteration in the NADPH content. Studying the role and mechanism of GOT1 in adipogenesis will provide a preliminary understanding of GOT1 in the distinct lipid depositions between the Mashen pigs and Large White pigs and the corresponding theoretical basis to improve the quality of pork.

## Figures and Tables

**Table 1:** sgRNA sequences

sgRNAs	DNA sequence of plus strand of sgRNA	DNA sequence of minus strand of sgRNA
sg1	accgCATTCGGTCCTATCGCTATT	aaacAATAGCGATAGGACCGAATG
sg2	accgAGAAGATCGTGCGAGTGACG	aaacCGTCACTCGCACGATCTTCT
sg3	accgACATTCGGTCCTATCGCTAT	aaacATAGCGATAGGACCGAATGT

The lower case denotes the sticky end sequence that is complementary to the *Bsmb* I cleavage site.

**Table 2:** Primer sequences

Gene	Primer sequence (5′-3′)	Length (bp)
*GOT1*	F: CATCCTGCGAGTCCTTTC	150
	R: CGGTCAGCCATTGTCTTC	
*MDH1*	F: TAAGGTTATCGTGGTGGG	124
	R: TGCTTTAGCTCGGTTGTG	
*PPARγ*	F: GCCCAGGTTTGCTGAATGTG	143
	R: CTGCCTGAGGTCCGTCATTT	
*AP2*	F: TGGGATGGGAAATCAACCACC	73
	R: CGTTCATGACACATTCCAGCA	
*18S*	F: CCCACGGAATCGAGAAAGAG	122
	R: TTGACGGAAGGGCACCA	

*GOT1*, glutamic-oxaloacetic transaminase 1; *MDH1*, malate dehydrogenase 1; *PPARγ*, peroxisome proliferator activated receptor gamma.
